# Cardiac Contractility Modulation Therapy and Device Algorithm–related Challenges

**DOI:** 10.19102/icrm.2025.16024

**Published:** 2025-02-15

**Authors:** Farman Ali, Khurram Arshad, Rabia Latif, Faizan Malik, Aman Ullah, Fnu Raheela, Sarita Rao, Sohail Hassan

**Affiliations:** 1 Department of Cardiology, Corewell Health Dearborn Hospital, Dearborn, MI, USA; 2 Department of Internal Medicine, McLaren Flint Hospital, Flint, MI, USA; 3 Department of Internal Medicine, Ameerudin Medical College, Lahore, Pakistan; 4 Department of Internal Medicine, SSM Health, St Louis University Hospital, St. Louis, MO, USA; 5 Department of Internal Medicine, Borgess Medical Center, Kalamazoo, MI, USA

**Keywords:** Algorithm problems, cardiac contractility modulation, case series, heart failure

## Abstract

Heart failure (HF) is a complex and potentially life-threatening medical condition posing significant risks to individuals. It is associated with substantial health care expenditures, considerable morbidity and mortality, and a decline in functional capacity and quality of life. Cardiac contractility modulation (CCM) has emerged as a promising device-based treatment for patients with HF with reduced ejection fraction (HFrEF). Studies have shown that CCM treatment in HFrEF patients can improve exercise tolerance and quality of life and reduce HF hospitalizations. As CCM therapy becomes a more prevalent treatment for HFrEF, the natural learning curve inherent in the use of new technologies necessitates broader provider training and careful patient follow-up. To that end, this article highlights the importance of developing a fundamental troubleshooting algorithm to help optimize the management of patients who have an implanted CCM device.

## Introduction

Heart failure (HF) is a major contributor to morbidity and mortality globally.^1^ Many patients remain symptomatic despite receiving guideline-directed medical therapy (GDMT) and have a high mortality rate mainly due to the limitations in medication up-titration. HF device therapy includes cardiac resynchronization therapy (CRT) for patients with a left ventricular ejection fraction (LVEF) of ≤35% and a comprehensive QRS complex (QRS duration ≥130 ms) and an implantable cardioverter-defibrillator (ICD) for patients with an LVEF of ≤35%. Many clinical studies have shown the efficacy of CRT in patients with HF with reduced ejection fraction (HFrEF).^2–5^ Most recent HF guidelines recommend the use of CRT in eligible patients. Unfortunately, not all patients with HFrEF have a prolonged QRS complex and hence are not candidates for CRT.

Cardiac contractility modulation (CCM) device–based therapy is a newer option for such patients. The Optimizer Smart Implantable Pulse Generator (IPG) (Impulse Dynamics, Marlton, NJ, USA) CCM device is being accepted into clinical practice for patients with HF, having been shown to improve peak VO_2_, the 6-min walk test, and overall symptoms of congestive HF (CHF). Further data from observational studies demonstrate improvements in ejection fraction (EF) and a decrease in HF hospitalization.^6–9^

Approved by the U.S. Food and Drug Administration, the device is a minimally invasive implantable system used in HF patients with EFs of 25%–45%, experiencing New York Heart Association (NYHA) class III symptoms and not receiving CRT despite being on GDMT. Patients receiving CCM therapy experience a better quality of life, characterized by reduced symptoms associated with CHF, such as shortness of breath and fatigue.^6–9^ The device is rechargeable, with a projected longevity of 20 years, with weekly charging sessions performed by the patient at home using an external charger (Guardio™; Impulse Dynamics), generally lasting an hour per session. At the start of each recharging period, the device performs a scaled-down “interrogation” through the external charger, which can generate alphanumeric alert codes on the patient interface indicating any change in integrity or performance.

## How cardiac contractility modulation works

The device is implanted similar to a pacemaker, with two bipolar active fixation leads positioned at the right ventricular (RV) septum (labeled local sense [LS] and RV, as described earlier), and delivers high-voltage non-excitatory biphasic electrical impulses with a wide pulse width during the absolute refractory period (systolic phase) to induce an acute augmentation of left ventricular (LV) contractile strength.^7^ After implantation of the CCM device, it is programmed and activated intraoperatively, so the patient can start receiving CCM therapy typically 5 h/day in equally spaced-out intervals, with a voltage between 4 and 7.5 V and aiming for a maximal percentage of CCM therapy delivery during these programmed windows.^8^

These leads are designated as V1 and V2 in the device header, with timing labels of “right ventricular” (RV) and “local sense” (LS) that can be assigned to either channel based on the pattern of sensed conduction (generally, the RV label should be applied to the lead sensing the earliest ventricular activation), regardless of the anatomical position of leads. Therefore, the vector of activation is established by assignment of the lead labels (ie, activation sensed on RV and then LS), and the timing between sensing of ventricular activation at each lead should be relatively consistent, regardless of the supraventricular rhythm, with a programmable LS alert window set to start based on RV activation at a nominal 30-ms duration. When a stable, predominant pattern of ventricular activation is sensed, this is programmed into the algorithm, and CCM therapy is delivered by the device on each beat matching this pattern during a scheduled therapy window. The current delivery varies from 4–7.5 V. Given this timing pattern, CCM signals do not start new contractions but induce an acute, mild augmentation of LV contractile strength during sensed contractions and more broadly impact the biology of the failing myocardium without increasing myocardial oxygen consumption.^10^

As CCM therapy evolves into mainstream therapy for HF, we see various challenges that highlight the importance of the troubleshooting algorithm required to manage these patients with implanted CCM devices. This article describes three cases, which may help the treatment algorithms and workflow for CCM patients. Consent for publication was obtained, in line with the Committee on Publication Ethics (COPE) best practice guidelines, and the individuals reported on are aware of the possible consequences of that reporting.

The following are unique considerations specific to the use of CCM device therapy in HF patients. At any visit where device functionality, patient safety, or clinical efficacy is of concern, the following should be assessed to ensure proper operation:

1. The CCM pulse signal falls within 40 ms after the R-wave and terminates before the S-wave (generally assessed in the surface limb lead with the largest R-wave amplitude, typically lead II).
2. The CCM therapy current falls between 15 and 30 mA (calculated based on the lead impedance values at a maximum 7.5-V output on each lead).
3. The CCM treatment schedule is between 5 and 7 h (based on indications), with the “extend on low CCM” feature enabled on applicable devices.
4. The percent of CCM therapy delivered is maximized, with a goal of ≥70% per scheduled session above.
5. Any concomitant dual-chamber CRT device has been assessed for and excludes atrial oversensing, which could contribute to inappropriate mode switching or atrial tracking, leading to unnecessary RV pacing.

This device is an adjunctive therapy used to augment cardiac contractility; if these device-specific parameters are within the acceptable range specified, and there are no patient alerts from the device charger, any further clinical concerns or symptoms should be considered within the context of the underlying HF and managed accordingly.

## Clinical cases reviewing device alerts

### Patient 1: Lack of therapy due to double counting of local sensed event

A 60-year-old woman with a past medical history (PMH) of hypertension, systolic HF, and status/post (s/p) percutaneous aortic valve replacement due to severe aortic insufficiency continued to experience NYHA class III symptoms despite being on GDMT. She was evaluated for CCM therapy and underwent CCM device implantation on March 22, 2022.

**Figure 1:**
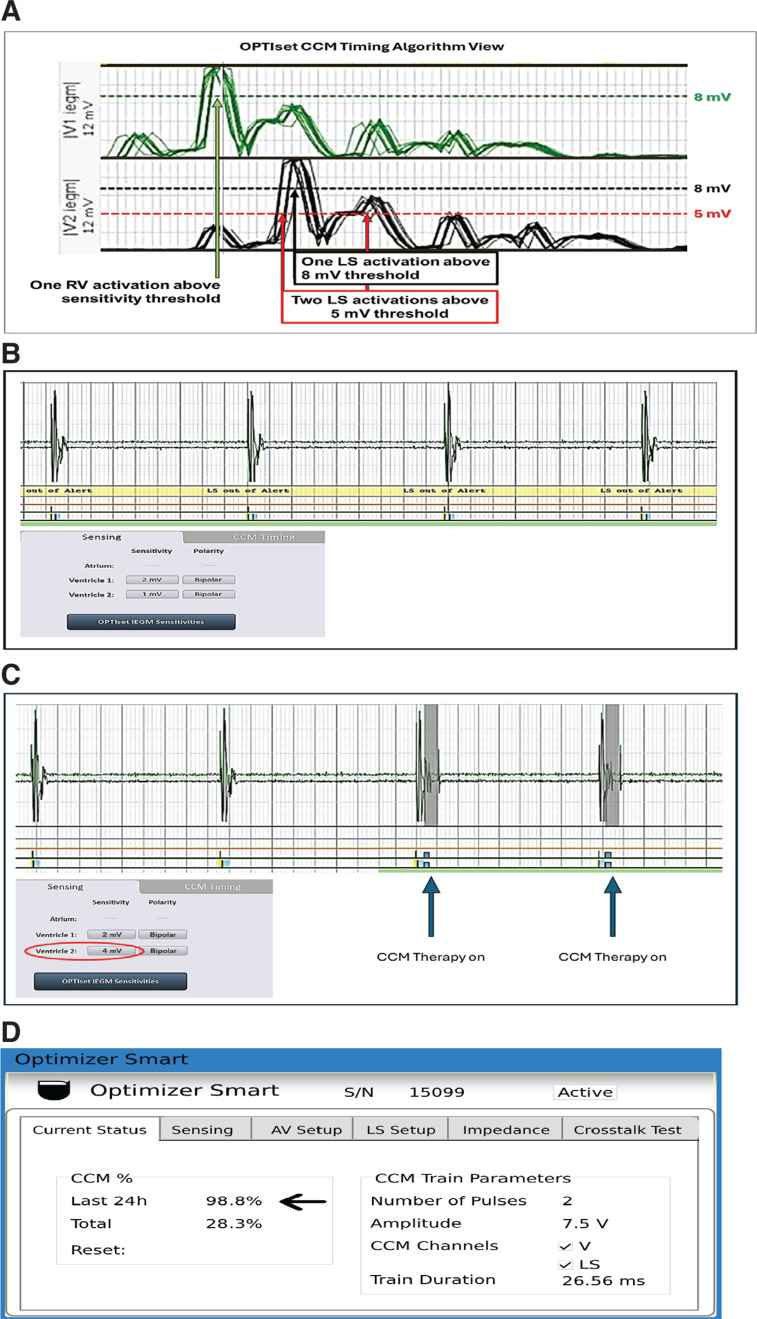
**A:** Illustration of the impact of local sense (LS) sensitivity changes from 5 to 8 mV with 1.0 mV depicting a double LS case. **B:** LS sensitivity programmed to 1 mV showing no therapy delivery at the end of each internal ventricular electrogram (LS) sensed by the device (red arrows). **C:** After the change to lower sensitivity to 4 mV, cardiac contractility modulation therapy. **D:** Illustration of 98.8% therapy delivery based on 4-month follow-up. *Abbreviations:* CCM, cardiac contractility modulation; LS, local sense; RV, right ventricular.

The patient experienced a continuous alert code 1 (lead impedance change), indicating a significant change in lead impedance. The device was interrogated, and the initial finding was that the presenting markers showed “double LS” on each beat, preventing therapy delivery. This condition results from detecting more than one above-threshold R-wave signal on the LS lead, which will result in the algorithm suspending delivery of CCM to ensure therapy is not delivered inappropriately. As the device was implanted, the percentage of therapy delivered was 9.8%, and total therapy was 27%. The RV electrode R-waves measured 2 mV with the RV sensitivity programmed to 1.0 mV. LS sensitivity was also programmed to 1.0 mV but with measured R-waves of 10 mV. LS oversensing was suspected, resulting in double LS and inhibition of therapy delivery. Programmed LS sensitivity was decreased from 1.0 to 4.0 mV, resulting in a single event on the LS lead and appropriate therapy delivery. With the return of therapy delivery, impedances were attainable, consistent, and stable: RV, 362; LV, 440. The pacemaker was also interrogated, and crosstalk testing was performed with no pacing inhibition or oversensing observed **(Figures 1A–1C)**.

On follow-up, the patient was found to have received 98.7% therapy since the last visit **(Figure 1D)**. The CCM lead impedances remained stable: RV, 370; LV 459. However, this alert is given priority over others like “low therapy percentage” (alert 4) and may be the presenting alarm if CCM therapy is suspended and automatic lead impedance testing cannot be performed by the device. The patient did not receive any further alerts during this period. The patient is clinically doing better on follow-up visits with improvement in her dyspnea and fatigue symptoms. Her brain natriuretic peptide (BNP) level improved from 944 to 213 ng/L.

### Patient 2: External magnet inhibiting therapy

**Figure 2:**
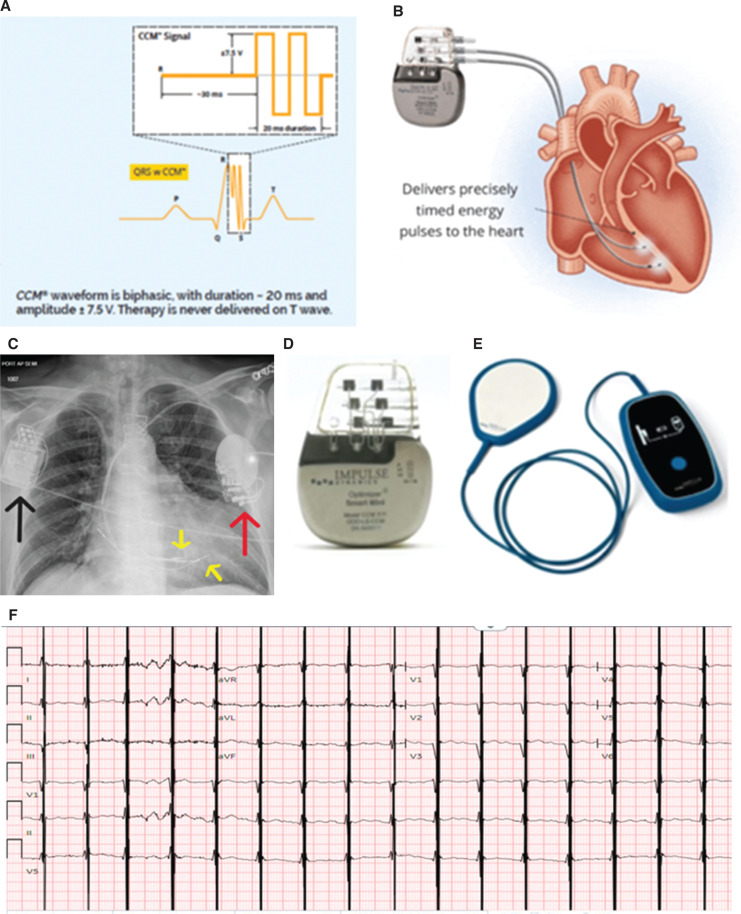
**A:** Mechanism of therapy delivery. **B:** Cardiac contractility module and lead placement in the heart. **C:** Chest X-ray with cardiac contractility modulation (CCM) Optimizer and implantable cardioverter-defibrillator in place. **D:** Image of a second-generation CCM Smart Optimizer. **E:** Vesta charger. **A, B, D, and E:** Images courtesy of Impulse Dynamics. **F:** Electrocardiogram of a patient actively receiving CCM therapy.

A 71-year-old man with a PMH of coronary artery disease (CAD), hypertension, s/p ICD placement in 2017, and hyponatremia continued to experience severe HF symptoms of NYHA class III while on GDMT. The patient was not a CRT candidate, as his electrocardiogram-based QRS duration was <130 ms. Hence, the patient underwent CCM device implantation on February 22, 2022 **(Figure 2C)**.

**Figure 3:**
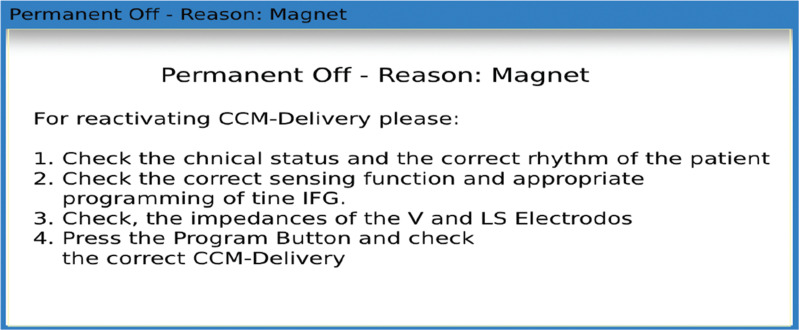
Cardiac contractility modulation device interrogation showing therapy disabled due to contact with the magnet. *Abbreviation:* CCM, cardiac contractility modulation.

The patient reported getting an alert code 2 (CCM inactive) for 2 months, but, unfortunately, the patient did not contact the company’s customer service or the device clinic. Device interrogation confirmed alert code 2, meaning therapy was disabled, with a dialogue box indicating that this was due to contact with a magnet **(Figure 3B)**.

Once CCM therapy is turned off by a magnet, there are two programmable options on the Optimizer Smart Mini device (released in the United States in 2022). In the earlier version of the device, the Optimizer Smart, therapy can be deactivated indefinitely by application of the magnet to the pulse generator, which must be reprogrammed manually for the reactivation of CCM therapy.

In the current version, the magnet application temporarily suspends for a 24-h cycle, at which point it will resume automatically. By default, the magnet application will suspend therapy for 24 h. In this case, before coming to the clinic, the patient was hospitalized due to CHF exacerbation at a local hospital for almost 3 weeks; unfortunately, the device implantation clinic was not informed about the admission. The company representative reactivated the device using the programmer.

The patient continued to experience symptoms of worsening HF and significant edema. At 4-week follow-up, the patient was receiving alert code 4 on the charger, suggesting the percentage of CCM therapy was low (programmable, with a nominal setting of <70%).

Device interrogation confirmed low therapy, and the patient received <1% therapy in the preceding 24 h. The LS lead sensing was found to be only 1.7 mV, with a programmed LS sensitivity of 3 mV, resulting in an “LS undersensing,” causing CCM therapy to be inhibited because of the inability to validate the ventricular activation pattern. The LS sensitivity was increased to 1 mV, at which time therapy resumed. Following this, the time from sensed activation on the RV lead to sensed activation at this new threshold on the LS lead (RV–LS timing) was found now to be 11–15 ms. At the prior threshold, the start of the 30-ms LS alert window was programmed at −2 ms from RV activation. This was moved to −6 ms from RV activation to ensure appropriate timing intervals for CCM therapy delivery. While continuing to monitor for appropriate CCM therapy delivery in the clinic, an “LS out of alert” started to occur due to intermittent sensing of LS signal at +30 ms post–RV activation. These were presumed to be changes due to pacing from the existing dual-chamber ICD. A Boston Scientific (Marlborough, MA, USA) programmer was used to interrogate the patient’s ICD, confirming that the RV–LS timing changes were due to intermittent RV pacing. We extended the atrioventricular delays of the ICD to limit unnecessary RV pacing and maintain consistent RV–LS timing. We also increased the hours of therapy per day to 7 h to compensate for any additional paced beats, which might reduce the overall percentage of CCM therapy delivered during a scheduled window. Upon completion of the interrogation, the patient was receiving 100% therapy.

On follow-up visit, the patient reported receiving alert code 1 (lead impedance change) since the last visit. Device interrogation showed that the patient had received only 50.4% therapy over the last 24 h, which correlated with an increase in RV pacing from the existing ICD to 50%. The RV-paced RV–LS timing was +29 ms, whereas the intrinsic RV–LS timing was +10 ms. Therefore, the starting point of the 30-ms LS sensing window (LS alert start) was changed from −6 ms from RV activation to +8 ms to try to capture both paced and sensed beats. The patient’s ICD was interrogated again to correlate what was being seen on the CCM device programmer. CCM therapy delivery was confirmed for both paced and sensed beats. Crosstalk testing was performed for both paced and sensed beats without oversensing or inhibition of the brady or tachy therapies from the ICD. The CCM lead impedances were stable now (RV, 380; LS, 377). We increased the low therapy alarm threshold from 70% to 80% and turned off the impedance alarm so that it would not supersede the low therapy alert. Doing so would alert us earlier if the therapy level were to fall below the 80% therapy alarm.

On a follow-up visit, device interrogation showed that the patient had received 99.3% therapy over the last 24 h; the impedance was stable: RV, 363; LS, 377. No programming changes were made. The patient was educated to charge the device twice per week for consistent therapy delivery for 7 h/day. Unfortunately, the patient’s hyponatremia and HF symptoms worsened, and he passed away.

### Patient 3: Cardiac contractility modulation therapy oversensed by atrial lead, leading to mode switching

A 71-year-old man with a PMH of hypertension, CAD s/p stenting, chronic obstructive pulmonary disease, HF with an EF of 30%, and s/p dual-chamber ICD placement underwent CCM device placement on August 28, 2023, as he was experiencing NYHA class III symptoms and was not a candidate for CRT given a normal QRS.

CCM device interrogation showed appropriate CCM function with appropriate amounts of therapy delivery. However, it was noted that the patient’s ICD device was sometimes oversensing CCM therapy on the atrial lead, causing inappropriate mode switching of the pacemaker. Attempts were made to decrease the atrial sensitivity of the pacemaker. Oversensing of CCM therapy was still occurring up to the point where the programmed sensitivity of the pacemaker was causing undersensing of intrinsic atrial activity.

**Figure 4:**
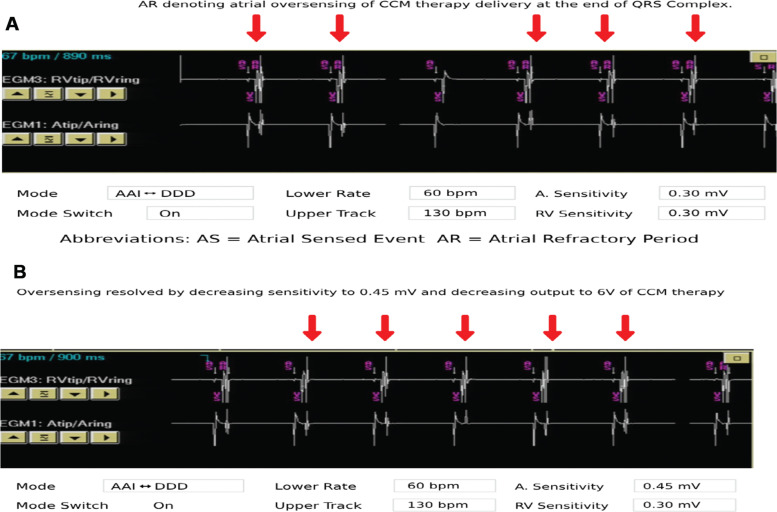
**A:** Atrial oversensing of cardiac contractility modulation (CCM) therapy by pacemaker is presented. **B:** Final permanent pacemaker (PPM) programming with atrial sensitivity set to 45 mV on PPM and CCM device output set to 6 V—no evidence of any oversensing of CCM therapy. *Abbreviations:* AR, atrial refractory period; AS, atrial sensed event; CCM, cardiac contractility modulation; PPM, permanent pacemaker; RV, right ventricular.

The pacemaker was returned to an accepted atrial sensitivity of 0.45 mV **(Figure 4A)**.

The possible options were (1) to manipulate the post-ventricular atrial blanking period to the point that sensed CCM therapy on the pacemaker was falling into the blanking period and (2) to adjust programmed CCM outputs. The second option was selected, and attention was paid to the patient’s CCM device to mitigate the issue. The CCM device was programmed at a maximum output of 7.5 V. The CCM output was decreased in half-volt (0.5 V) increments to the point where the pacemaker was no longer oversensing the CCM therapy, and it occurred at an output of 6 V on the CCM device. A current calculation was performed to ensure that the patient still received a therapeutic current of delivered energy. At 7.5 V, the patient had been receiving 35.40 mA of current. At the final programmed output of 6 V **(Figure 4B)**, the patient received 28.32 mA of current. The current therapeutic range of CCM therapy is estimated to be between 15 and 50 mA, without a correlation demonstrated to date of higher or lower current and clinical outcomes.

This programming change of the CCM device allowed for continued therapeutic delivery of CCM therapy while preventing any oversensing of CCM by the patient’s pacemaker and without manipulating the final programming of the patient’s pacemaker, ensuring appropriate sensing of intrinsic atrial activity. The patient was being followed up in the office regularly, and his BNP improved from 1035 to 140 ng/L, along with improvement in his dyspnea and leg edema.

## Discussion

Optimizer Smart (Impulse Dynamics) device–based CCM therapy is a relatively newer approach for HF patients, and health care providers need to familiarize themselves with this evolving technology for appropriate device management and patient care. As one of our patients had CCM therapy turned off during hospitalization, likely due to contact with a magnet, this emphasizes the need for greater awareness of this newer technology. At the same time, patients need more education and clear instructions on when to reach out to the device clinic and company customer service when receiving alerts on their charger. The manufacturer is currently working on remote follow-up of these devices.

Eight alert codes can appear on the Optimizer charger to alert the patient when IPG is being charged **(Table 1)**.

**Table 1:** Charger Error Codes—Optimizer Smart

Numeric Code	Code Description	Source	IPG Rechargeable?	Suggested Action
0	IPG deactivated	EMI or internal software conflict	No	Prompt follow-up for assessment and reactivation
1	Lead impedance change	Normal post-implant changes versus lead malfunction	Yes	Prompt follow-up for assessment if message persistent
2	CCM inactive	Exposure to strong magnetic field or high-current condition with low impedance	Yes	Prompt follow-up for impedance evaluation
3	CCM delivery not programmed	CCM turned off or in “standby”	Yes	Prompt follow-up for reactivation
4	% CCM therapy low	Sensing changes, arrhythmia, or lead malfunction	Yes	Prompt follow-up for arrhythmia management, mode, sensing, impedance, and/or detection window adjustment
5	Optimizer Smart temperature issue	Febrile illness or abnormal IPG/charger interaction	Not suggested	Pause charging, prompt follow-up, physical exam
6	Internal charger fault	Charger malfunction	No	Prompt follow-up for charger replacement
7	IPG (not Optimizer Smart device)	Wrong-generation Optimizer charger	No	Re-educate appropriately matched charger use
8	Battery depleted	Battery discharged completely or has operation interference	Not suggested	Pause charging, prompt follow-up

*Abbreviations:* CCM, cardiac contractility modulation; EMI, electromagnetic interference; IPG, Implantable Pulse Generator.
